# Tranexamic acid in spontaneous intracerebral hemorrhage: a meta-analysis

**DOI:** 10.1186/s40001-025-02385-x

**Published:** 2025-02-25

**Authors:** Yu Chang, Pei-Chun Lai, Chih-Yuan Huang, Junmin Song, Kuan-Yu Chi, Hsiang-Yi Hung, Yen-Ta Huang

**Affiliations:** 1 Department of Surgery, National Cheng Kung University Hospital, College of Medicine, National Cheng Kung University, Tainan, Taiwan; 2 Education Center, National Cheng Kung University Hospital, College of Medicine, National Cheng Kung University, Tainan, Taiwan; 3 Department of Medicine, Jacobi Medical Center, Albert Einstein College of Medicine, Bronx, NY USA; 4 Department of Neurosurgery, Hualien Tzu Chi Hospital, Buddhist Tzu Chi Medical Foundation, Hualien, Taiwan; 5 Institute of Medical Sciences, Tzu Chi University, Hualien, Taiwan

## Abstract

**Background:**

Spontaneous intracerebral hemorrhage (sICH) is a critical and disabling form of stroke and accounts for an obvious number of stroke-related deaths and disabilities globally. Hematoma growth is a key target for therapeutic intervention because of its association with poor outcomes. Recently, the STOP–MSU trial showed that intravenous tranexamic acid (TA) did not reduce hematoma growth or improve clinical outcomes when administered within 2 h of intracerebral hemorrhage symptom onset. This study aims to evaluate the efficacy of TA in reducing hematoma growth and improving clinical outcomes in patients with spontaneous sICH by incorporating the findings from the latest STOP–MSU trial and consolidating past research to clarify the overall efficacy and safety of TA on sICH.

**Methods:**

A systematic review and meta-analysis were conducted according to the Cochrane Handbook for Systematic Reviews of Interventions and PRISMA guidelines. We included randomized controlled trials (RCTs) comparing TA to placebo in adult patients with sICH. Databases such as PubMed, Medline, and Cochrane were searched up to May 2024. Key outcomes analyzed included hematoma expansion, mortality within 90 days, thromboembolic events, and favorable functional outcomes. Data were pooled using a random-effects model and analyzed using the “metafor” package in RStudio.

**Results:**

Five RCTs involving 1419 patients were included. The meta-analysis showed no significant difference in hematoma expansion (odds ratio [OR] 0.87, 95% confidence interval [CI] 0.74–1.03), mortality within 90 days (OR 1.03, 95% CI 0.86–1.24), thromboembolic events (OR 1.07, 95% CI 0.69–1.64), and favorable functional outcomes (modified Rankin Scale of 0–2 at 90 days; OR 1.04, 95% CI 0.88–1.22) between the TA and placebo groups.

**Conclusions:**

TA does not significantly reduce hematoma growth or improve clinical outcomes in patients with sICH. Despite its affordability and availability, the routine use of TA in sICH is not supported by current evidence.

## Introduction

Spontaneous intracerebral hemorrhage (sICH) is one of the most lethal and disabling forms of stroke [1] and accounts for approximately 3.4 million incident cases globally each year [2]. Despite its lower incidence than ischemic stroke, sICH results in a comparable number of annual deaths and poses a higher burden of disability [3]. Effective evidence-based therapies for sICH remain extremely limited, with hematoma growth identified as a critical therapeutic target because of its association with increased functional disability and mortality [4]. Tranexamic acid (TA), an antifibrinolytic agent [5], has gained increasing research interest to explore whether it inhibits hematoma growth in sICH [6, 7].

A meta-analysis by Xiong et al. [8] included 25 randomized controlled trials (RCTs) and found that TA significantly inhibited hematoma growth in patients with sICH and traumatic brain injury (TBI). However, distinguishing between sICH and TBI is important. A new trial (STOP–MSU) [9] showed that intravenous TA did not reduce hematoma growth when administered within 2 h of sICH symptom onset. The study did not observe effects on other imaging endpoints, functional outcomes, or safety. Given these mixed results, we plan to conduct an updated meta-analysis to investigate the efficacy of tranexamic acid, specifically in sICH, including the latest trial results.

## Methods

We conducted a systematic review and meta-analysis in accordance with the Cochrane Handbook for Systematic Reviews of Interventions [10]. Our findings were presented following the Preferred Reporting Items for Systematic Reviews and Meta-Analyses (PRISMA) guidelines. The study was registered on the INPLASY platform (202,450,025).

### Study selection

The first two authors independently searched electronic databases, including PubMed, Medline, and Cochrane, from January 1900 to May 2024, to identify relevant studies using the keywords (tranexamic acid OR transamine OR TXA) AND ((intracranial hemorrhage OR intracerebral hemorrhage OR ICH OR brain hemorrhage OR cerebral bleeding OR intraparenchymal hemorrhage OR intracranial bleeding). Only articles published in English were included. In cases of discrepancies, a consensus was reached with senior reviewers.

### Eligibility criteria

The articles included in this analysis must meet several specific criteria. They should be RCTs involving adult patients over the age of 18 years who have primary sICH. These studies must compare the administration of TA with a placebo and assess at least one clinical outcome between the two groups.

### Data extraction

First two authors independently extracted relevant information from eligible articles. Any discrepancy was addressed by reaching a consensus with a senior reviewer.

### Quality assessment

First two authors independently conducted a critical appraisal of the included trials using the Cochrane Risk of Bias 2.0 (ROB 2.0) tool [11]. Any discrepancies were resolved by consulting a third senior investigator.

### Statistical analysis

Meta-analysis was conducted using RStudio with the “metafor’ package [12]. The primary outcomes are hematoma expansion and the proportion of favorable functional outcomes between the TA group and the placebo group. The secondary outcomes include mortality within 90 days and the incidence of thromboembolic events. For these categorical variables, we extracted the total number of participants in each group and the number of participants who experienced mortality and favorable outcomes from each article. The data were pooled using a random-effects model with Mantel–Haenszel method. The pooled odds ratio (OR) was presented with 95% confidence intervals (CIs) and *p* values. Heterogeneity was assessed using the I^2^ statistic, with *I*^*2*^ values of less than 25%, between 25 and 50%, and greater than 50%, indicating low, moderate, and high heterogeneity, respectively [13].

## Results

### Included studies

**Fig. 1:**
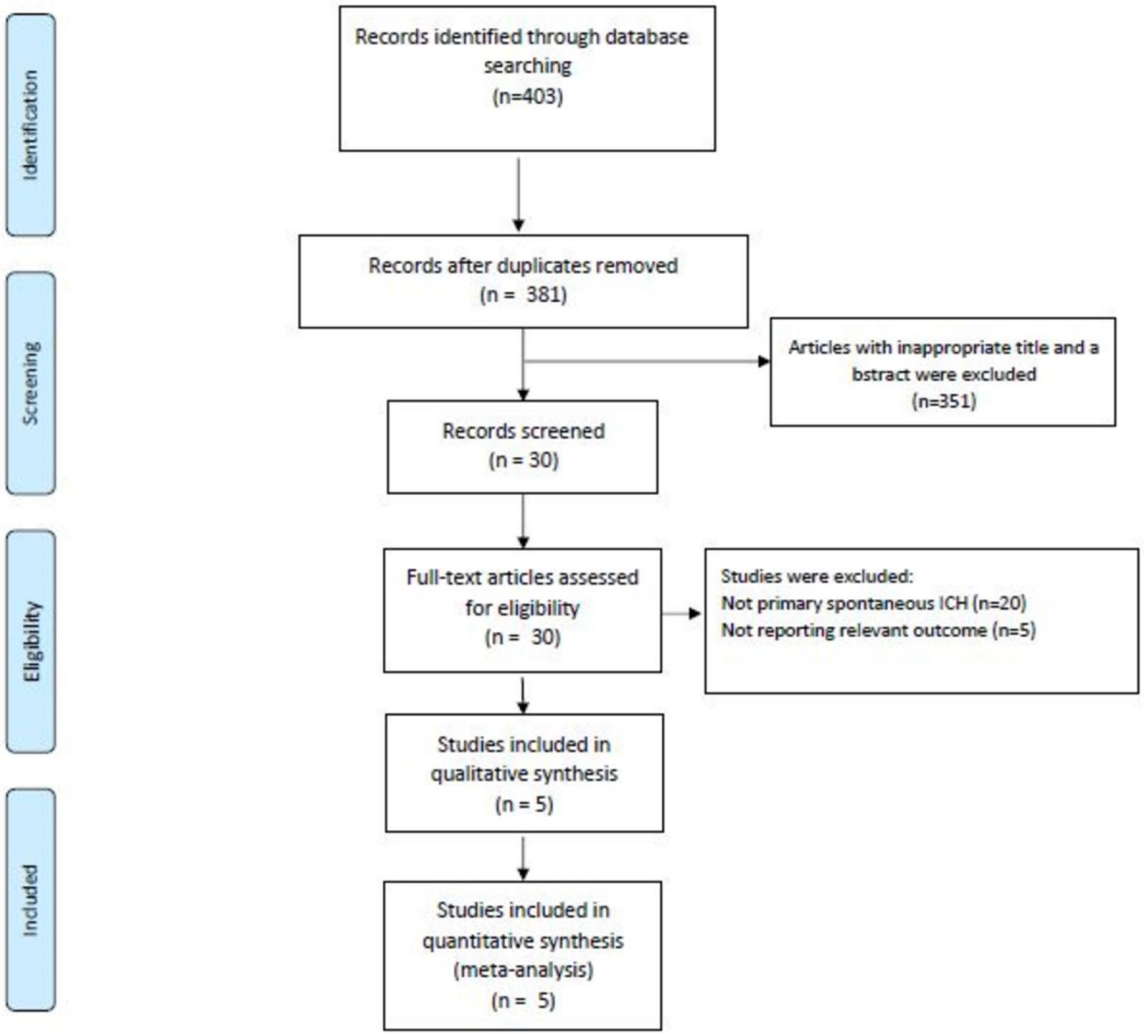
PRISMA diagram for study selection

Our search strategy identified 403 references from the electronic databases, with 30 studies selected for full-text inspection. Ultimately, we included the latest STOP–MSU [9] trial along with four additional RCTs [6, 7, 14, 15] (Fig. 1). The characteristics of the five included studies are summarized in Table 1. All enrolled studies reported the same dose of TA prescribed. The TA group included a total of 1,419 participants, while the placebo group had 1,402 participants. Of these, 57.0% of the TA group and 57.9% of the placebo group were male. The average ages were 68.2 years for the TA group and 67.4 years for the placebo group.

**Table 1:** Characteristics of the included studies

Study	Inclusion criteria	Exclusion Criteria	Tranexamic Acid Administration	Tranexamic Acid Group	Control Group
Liu 2021 (TRAIGE)	Acute supratentorial intracerebral hemorrhage, indication of hemorrhage expansion on imaging, treatable within 8 h of symptom onset	ICH secondary to tumor, trauma, aneurysm, vascular malformation, hemorrhagic conversion of ischemic stroke, venous sinus thrombosis or central nervous system infection, use of oral anticoagulant therapy with abnormal laboratory values, infratentorial ICH, GCS score < 8, ICH volume > 70 mL, hemorrhage expanding to fill one side of the lateral ventricle or more than half of both lateral ventricles, clinical history or current evidence suggestive of venous or arterial thrombotic events	Intravenous tranexamic acid 1 g in 100 mL 0.9% NaCl over 10 min followed by 1 g in 250 mL 0.9% NaCl infusion over 8 h	*N* = 89	*N* = 82
Sprigg 2018 (TICH2)	Adults with acute intracerebral hemorrhage, admitted within 8 h of stroke symptom onset	ICH secondary to anticoagulation, thrombolysis, trauma, or known underlying structural abnormality; prestroke dependence (mRS > 4); life expectancy < 3 months; GCS score < 5	Intravenous tranexamic acid 1 g in 100 mL normal saline 0.9% over 10 min, followed by another 1 g in 250 mL normal saline 0.9% over 8 h	*N* = 1161	*N* = 1164
Yassi 2024 (STOP–MSU)	Adults with acute spontaneous intracerebral hemorrhage confirmed on non-contrast CT, treated within 2 h of stroke symptom onset	Baseline Glasgow Coma Scale score < 8, brainstem hemorrhage, hematoma volume > 70 mL, recent use of anticoagulants, hemorrhage due to trauma or other secondary causes	Intravenous tranexamic acid 1 g over 10 min followed by 1 g over 8 h	*N* = 103	*N* = 98
Meretoja 2020 (STOP–AUST)	Aged 18 years or older, acute intracerebral hemorrhage with a spot sign on CT angiography, treatable within 4.5 h of symptom onset and within 1 h of CT angiography	Glasgow Coma Scale score < 8, contraindications for antifibrinolytic therapy, ICH > 70 mL, brainstem hemorrhage, ICH secondary to trauma, aneurysm, vascular malformation, hemorrhagic transformation of ischemic stroke, cerebral venous thrombosis, thrombolytic therapy, tumor, or infection, thromboembolic events in the past 12 months, planned surgery within 24 h, use of anticoagulation agents, pregnancy, concurrent use of hemostatic agents	Intravenous tranexamic acid 1 g in 100 mL 0.9% NaCl over 10 min followed by 1 g in 500 mL 0.9% NaCl infusion over 8 h	*N= *50	*N* = 50
Sprigg 2013	Adult patients with acute (< 24 h after ictus) spontaneous ICH	Secondary ICH (anticoagulation, known vascular malformations), previous venous thromboembolic disease, recent (< 12 months) ischemic events, renal impairment, and pregnancy or breast feeding	Intravenous tranexamic acid 1 g loading dose infusion for 10 min followed by a 1 g infusion for 8 h	*N* = 16	*N* = 8

CT, Computed Tomography, GCS, Glasgow Coma Scale, ICH, Intracerebral Hemorrhage, mRS, Modified Rankin Scale

### Risk of bias assessments

**Fig. 2:**
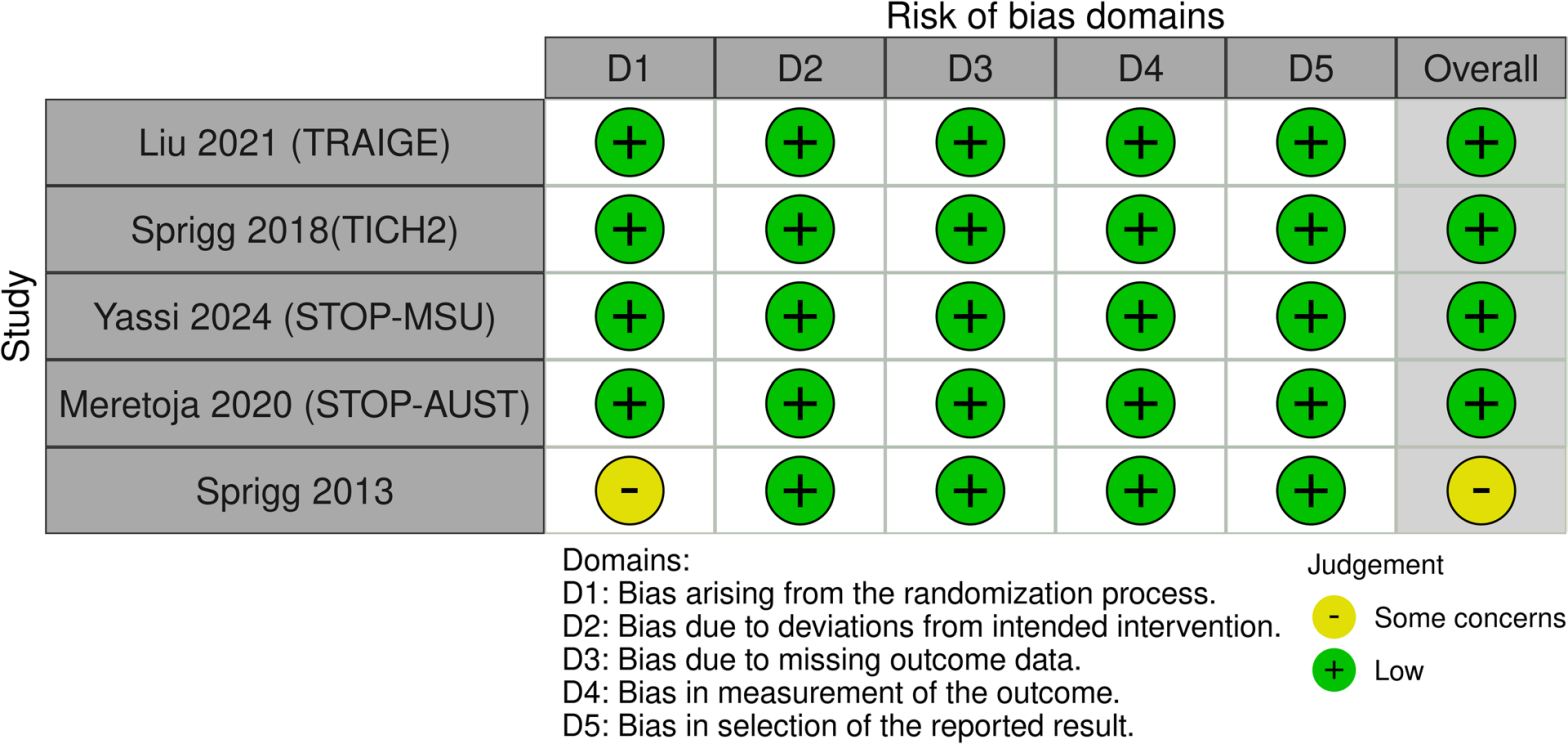
Quality assessment of included studies using ROB 2.0 tool

Using the ROB 2.0 tool for quality assessment, we found that the overall risk of bias was low in four of the included trials. However, one trial was classified as having some concerns due to unclear randomization processes. The detailed assessment is presented in Fig. 2.

### Pooled analysis

**Fig. 3:**
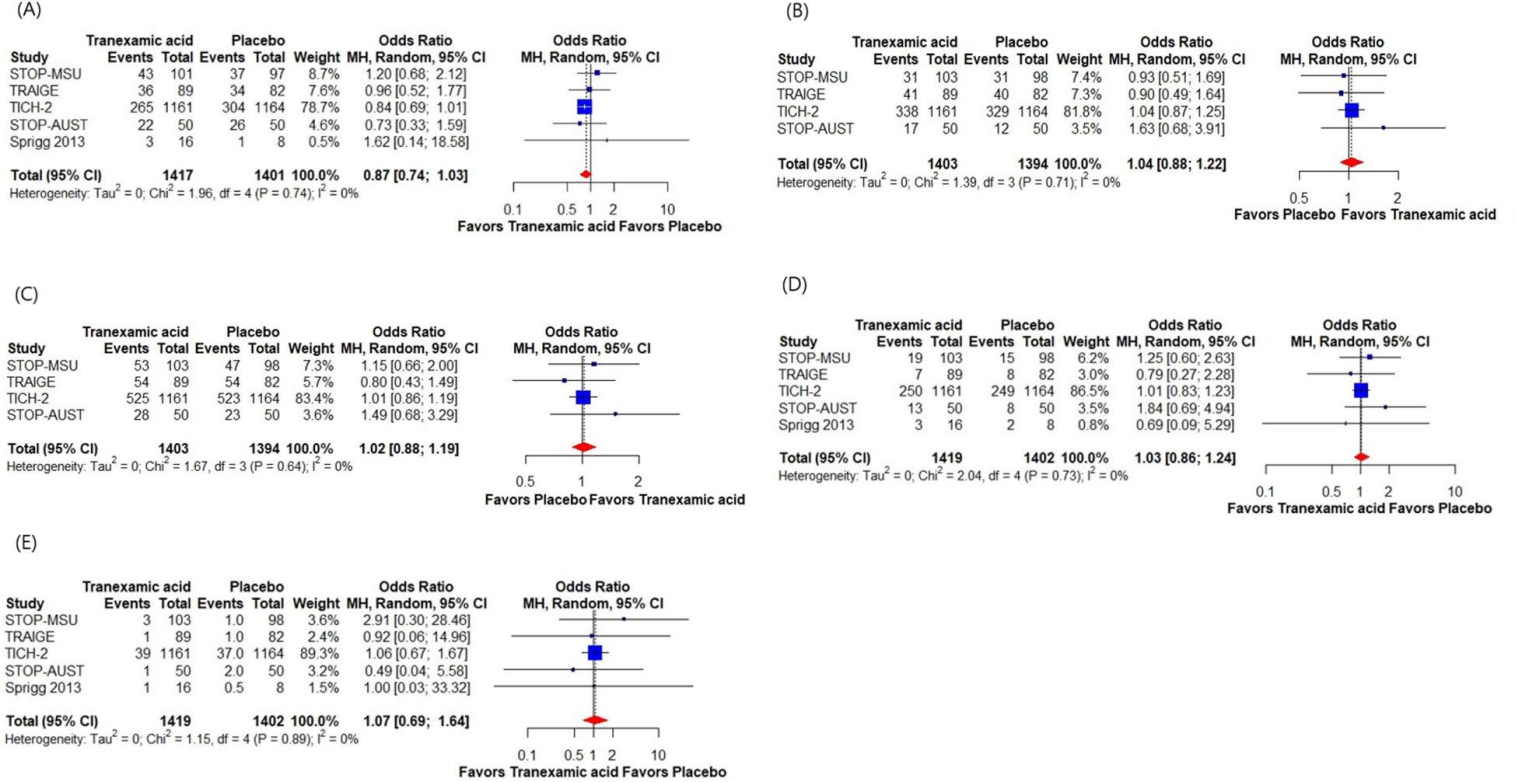
Pooled odds ratios for various outcomes in tranexamic acid versus placebo. **A** Hematoma expansion **B** Favorable functional outcomes (mRS 0–2 at 90 days). **C** Favorable functional outcomes (mRS 0–3 at 90 days). **D** Mortality within 90 days. **E** Thromboembolic events. CI, confidence interval; mRS, modified Rankin Scale; OR, odds ratio

The meta-analysis pooled results from five studies comparing TA to placebo in patients with sICH yielded several key findings (Fig. 3). For hematoma expansion, the pooled OR from five studies was 0.87 (95% CI 0.74–1.03, I^2^ = 0%, Fig. 3A), indicating no significant difference between the TA and placebo groups. In terms of favorable functional outcomes, assessed by the modified Rankin Scale (mRS), the OR values from four studies were 1.04 (95% CI 0.88–1.22, I^2^ = 0%, Fig. 3B) for mRS 0–2 at 90 days and 1.02 (95% CI 0.88–1.19, I^2^ = 0%, Fig. 3C) for mRS 0–3 at 90 days. These results suggest no significant difference in functional outcomes between the groups. Mortality within 90 days, pooled from four studies, had an OR of 1.03 (95% CI 0.86–1.24, I^2^ = 0%, Fig. 3D), indicating lack of significant difference between the TA and placebo groups. Thromboembolic events, pooled from four studies, showed an OR of 1.07 (95% CI 0.69–1.64, I^2^ = 0%, Fig. 3E), indicating no significant difference between the groups. The pooled results are summarized in Table 2.

**Table 2:** Summary of pooled results

Outcome	Number of Studies Included	Odds Ratio (95% CI)	I-square (%)
Hematoma expansion	5	0.87 (0.74–1.03)	0
mRS 0–2 at 90 days	4	1.04 (0.88–1.22)	0
mRS 0–3 at 90 days	4	1.02 (0.88–1.19)	0
Death within 90 days	4	1.03 (0.86–1.24)	0
Thromboembolic event	4	1.07 (0.69–1.64)	0

CI, confidence interval, mRS modified Rankin Scale

## Discussion

sICH is a form of stroke characterized by bleeding within the brain tissue itself and accounts for a substantial proportion of stroke-related mortality and morbidity [16]. Historically, the management of ICH has been challenging, with limited effective therapeutic options. AHA/ASA guidelines [17] for the spontaneous ICH management suggest that minimally invasive approaches for supratentorial and intraventricular hemorrhages may reduce mortality, though functional outcome evidence remains neutral. For cerebellar hemorrhage, surgical evacuation is recommended for volumes > 15 mL or cases with neurological deterioration. The guidelines also emphasize the complexity of life-sustaining treatment decisions, stressing that these should be individualized, patient-centered, and not solely based on severity scales.

The STICH and STICH II trial [18, 19] investigated the role of early surgical intervention in ICH but found no significant benefit in terms of survival or functional outcomes; the results highlight the need for alternative treatment strategies. This gap in effective treatments led researchers to investigate whether medications could prevent hematoma expansion, prompting the emergence of trials examining the efficacy of TA.

Although the latest meta-analysis by Xiong [8] suggested that TA can significantly reduce the risk of intracranial hemorrhage growth, it included a significant number of trauma-related hemorrhage cases, which differ in nature from sICH. A closer review of trials focused on sICH, such as STOP–AUST [14] and TICH-2 [7], found no evidence that TA prevents sICH growth, although the treatment was safe with no increase in thromboembolic complications. The most recent STOP–MSU trial [9] also found no benefit. Our meta-analysis supports these findings and provides comprehensive verification of the results. Despite the absence of statistical heterogeneity (I^2^ = 0%) in our meta-analysis, the inclusion timing in each study varied: the TICH-2 trial [7] included patients treated within 8 h of stroke symptom onset, the STOP–MSU trial included those treated within 2 h, and the STOP–AUST trial [14] included patients with a CT spot sign treatable within 4.5 h. These variations in inclusion timing could impact the results and should be considered when interpreting the overall effectiveness of TA in sICH.

Although TA is an inexpensive and readily available drug, it does not improve clinical outcomes in patients with sICH. Therefore, its administration should be carefully considered. Beyond the risk of thromboembolism [20], another potential complication that has not been adequately addressed in these trials is seizures. TA has been associated with an increased risk of seizures, particularly when administered at high doses [21]. The proposed mechanism involves the inhibition of gamma-aminobutyric acid and glycine receptors in the central nervous system, which can lead to neuronal hyperexcitability and, subsequently, seizures [22]. This potential side effect is particularly concerning in the context of sICH where maintaining neurological stability is critical [23]. Future research should not only focus on the efficacy of TA but also on its safety profile, including the risk of seizures, to ensure a comprehensive understanding of its benefits and risks in treating sICH.

While our meta-analysis provides valuable insights into the efficacy and safety of TA in the treatment of sICH, several limitations must be acknowledged. First, the included studies exhibited variability in their inclusion criteria and the timing of TA administration, ranging from within 2 h up to 8 h of symptom onset. This heterogeneity in treatment windows could influence the outcomes and complicate direct comparisons. Second, despite our comprehensive search strategy, focusing exclusively on RCTs for sICH while ensuring a homogeneous patient population, resulted in a small number of included studies. As such, the statistical power was limited to detect significant differences. Finally, our analysis primarily addressed commonly reported outcomes, such as hematoma expansion, mortality, and thromboembolic events, but did not extensively consider other important clinical complications, such as seizures. These complications could significantly impact the overall safety and efficacy of TA in this patient population.

## Conclusions

Our meta-analysis results align with the latest international, double-blind, randomized, phase 2 trial, which consistently showed that intravenous TA did not reduce hematoma growth and did not significantly improve long-term clinical outcomes. Hence, TA acid should not be used routinely in sICH.

## Competing interests

The authors declare no competing interests.

## Data Availability

No datasets were generated or analysed during the current study.
